# The Influence of Geographical Environment on Public Social Trust: What Role Do Tourism Activities Play?

**DOI:** 10.3390/bs14030218

**Published:** 2024-03-07

**Authors:** Yang Gao, Zhenbin Zhao, Yaofeng Ma, Ping He, Yuan Li

**Affiliations:** 1 School of Geography and Tourism, Shaanxi Normal University, Xi’an 710119, China; 2 Centre for Mental Health Education, Xi’an University of Science and Technology, Xi’an 710119, China; 3 School of Psychology, Shaanxi Normal University, Xi’an 710119, China

**Keywords:** social trust, tourist reception, tourist supply, rice theory, pathogen stress theory, per-capita GDP

## Abstract

Social trust is derived from the interaction of environmental and social factors, which has important significance for the sustainable development of society and social governance. In particular, in the post-pandemic era, tourist activity will receive special attention in terms of its role in the development of the public’s social trust. On the basis of the sample of big data, this research takes China as an example to study the influences of different geographical and environmental elements on individuals’ social trust as well as the common role played by the tourist activity. The research showed that the geographical environment and tourism activities have interacting effects on public social trust. This influencing mechanism is specifically manifested as the rice-growing ratio and tourist reception level can have interacting effects on the social trust of the residents in a tourist destination; pathogen stress and tourist supply level can exert interacting effects on the social trust of the residents in an area from which tourists originate; and economic development and tourist reception level can have interacting effects on the social trust of the residents in a tourist destination. By doing so, this research provides theoretical support and practical suggestions for the recovery of the public’s social trust from the perspective of tourism geography in the post-pandemic era.

## 1. Introduction

Social trust is an important research issue in the field of community psychology. It is of great significance for the good operation of society and the positive development of individuals [1,2,3,4]. Social trust can be divided into specific and generalized trust. The former refers to an individual’s familiarity with and trust in a specific person, and the latter refers to an individual’s trust in the majority of strangers [5,6]. In modern society, generalized trust is deemed more important than specified trust [7], and it is conceptualized as a type of social capital in a sense and can be used and translated into other forms of capital, such as economic and intellectual capital [8,9,10,11]. At a macro level, generalized trust is considered helpful for a range of reasons, such as improving the quality of government management, driving economic growth, and boosting an individual’s subjective well-being, social cohesion, and citizen participation [12,13,14,15]. At a micro level, generalized trust has been proven to enhance people’s sense of fairness, social relations, and positive work attitudes and behaviors [16,17].

This study chooses generalized trust as the conceptual basis. Trust can exert these significant influences for a number of reasons—it “saves” the cognitive resources of an individual, constructs mental representations of the environment for an individual, and mobilizes an individual’s willingness to cooperate with other people [18,19]. In existing studies, social trust is regarded as an important measure of community cohesion, and enables a community to thrive [20,21]. Stolle [22] distinguishes two major areas that may benefit from a high level of trust: in the social field, social trust can boost tolerance and acceptance within a group, thus enabling a more diverse community to be built; in the political field, trust can be translated into a stronger driving force for citizens to participate in political affairs. For these reasons, the question of how to promote public social trust has become an important research problem in the domain of social governance [23]. This study described in this paper uses generalized trust as its conceptual basis and aims to investigate the interacting effects of residential activities and tourism activities on public social trust. General social trust can be measured by both positive and negative dimensions. Positive dimensions focus on the question “In general, do you think that most people can be trusted?”. Negative dimensions focus on the view that “Others will find ways to take advantage of you if you are not careful” [24,25]. For the first question, if an individual’s score exceeds the median, it indicates a certain level of social trust; if it is below the median, it indicates a lack of social trust. For the second question, the opposite applies.

Through a consideration of existing literature, it can be found that there is a close logical connection between individual residential activities and tourism activities in time and space [26,27,28]. Regarding geographical factors, residential activities are those conducted by an individual in a specific space for a long period of time, while tourism activities are the activities of an individual for a short period of time [29,30,31]. In terms of time, residential activities may last as long as several years or decades, while tourism activities may last only for several days. In terms of space, residential activities are concentrated in an area defined by homes and workplaces and vary in size depending on the size of the city. Tourism activities refer to leisure activities at least 75 km away from a person’s home. From the perspective of the human–land relationship, residential activities and tourism activities repeat themselves alternately in the life of an individual.

Both residential and tourism activities have subtle effects on mentality and behavior—most significantly the level of individual social trust. Many studies have found that an individual’s geographical environment and place of residence can influence their social trust, especially the latitude [32,33,34,35,36]. For instance, the difference in climate at different latitudes has a direct influence on the mentality and behavior of an individual, but also an indirect influence through social and cultural factors such as agriculture, economy, and effect on health [37,38,39]. Many studies in the field of tourism have also found that tourism activities have an enduring and stable influence on the mentality and behavior of an individual. For example, researchers have found that tourism activities can not only drive economic growth and improve quality of life but also reinforce positive emotions and enhance national identity [39,40,41,42]. Promoting the deep involvement of tourism activities in national social governance has become a major trend in recent years.

For this study, individual residential and tourism activities are independent of each other but are connected in time and space, so cannot be simply separated. As regards their differences, tourism activities have more diverse temporal and spatial representations because of different travel times and destination choices. They also have strong situational attributes because they are not based in an individual’s area of residence [31,43]. For these reasons, it is impossible to directly draw cross-situational inferences about the mentality and behavior of individuals when they are tourists. For their connection, human mentality and behavior are consistent and durable. Although residential and tourism activities are relatively independent in time and space, individual mentality and behavior are connected in these two situations, with relative yet non-absolute inertia and stability [26,27,28].

In summary, the study of the law of individual mentality and behavior needs to take the situationality of residential and tourism activities into consideration, as simply separating them is unreasonable and inaccurate. To better study the significant theoretical problem of the factors and mechanisms influencing public social trust, this study investigated the interacting effects of residential and tourism activities on public social trust, based on a review of existing independent research on residential and tourism activities.

## 2. Theoretical Framework and Hypotheses

### 2.1. Geographical Environment and Social Trust

In recent years, the research direction of latitudinal psychology has received increasing attention. Researchers are beginning to realize that the natural environment not only affects an individual’s physical state but also has diverse effects on their psychological state. In particular, temperature, agriculture, and pathogen transmission in their geographical environment have been found to significantly affect an individual’s psychological state [39].

#### 2.1.1. Clash (Class, Aggregation, and Self-Control in Humans) Theory

The primary natural environmental difference between different latitudes is their climate. Latitude directly influences the sunlight in different areas, leading to differences in local temperature and other aspects of climate. Researchers hold that climate is an important environmental factor that influences individual social trust [33,34,38,44,45,46]. The clash theory proposed by Van Lange et al. [39] suggests that colder winters and greater seasonal changes in temperature in high latitudes require individuals to make a more thorough annual plan. For example, people will store food and fuel for winter in advance. Thus, individuals need to employ a long-term and reasonable goal-oriented life strategy. In contrast, individuals who live in areas with more comfortable temperatures and smaller temperature differences throughout the year will pay more attention to the present in their lives and do not need to plan and prepare ahead for the rest of the year. As a result, they habitually pay less attention to self-control. This short-term, goal-oriented life strategy and the habitual lack of self-control will give rise to a more aggressive and violent social climate [33,34]. Consistent with the findings of clash theory, numerous studies have found that intra- and extra-group hostility, violent crimes, family conflicts, press suppression, political oppression, and legal discrimination reach their peaks near the equator and gradually decrease in higher latitudes [47,48,49].

#### 2.1.2. Rice Theory

Climate conditions in different latitudes also result in differences in agriculture. In China, as the latitude increases, the rice-growing ratio shows relatively regular changes—more crops, such as wheat, are cultivated in the north, and more rice is planted in the south. The difference in farming also influences the level of social trust of individuals in different areas. The rice theory proposed by Talhelm et al. [50]. suggests that, historically, rice-growing communities have stronger reciprocity in life and work than wheat-growing communities. To manage the irrigation network, the residents of rice-growing communities must coordinate the use of water and share infrastructure. This has generated a social culture in which people are mutually dependent on a close-knit social network [51,52]. Therefore, the closeness of community relations has become a major sociocultural difference between residents in rice- and wheat-growing areas [53,54,55,56,57].

#### 2.1.3. Pathogen Stress Theory

Different latitudes also have different epidemic infection rates because of their climates. The infection rate of epidemics, such as influenza, varies significantly by latitude. Over a long period, the incidence of epidemics has a significant influence on the level of social trust of residents. Pathogen stress theory suggests that diseases transmitted between humans manifest in the culture as, for example, collectivism, exclusivity, and ethnocentrism [58,59]. The reason behind this phenomenon is that residents in areas with high levels of pathogens try to reduce the risk of infection by reducing social interaction with strangers. Over time, such lifestyle habits may have a subtle impact on their social trust. The reason behind this phenomenon is that less social interaction in areas with a higher level of pathogen stress helps avoid infections by reducing contacts and interactions with strangers, and vice versa.

### 2.2. Economic Development and Social Trust

The level of economic development is a comprehensive concept, which is often uniformly measured by the Gross Domestic Product (GDP) index. The GDP index includes various measurement methods, such as regional and per-capita GDP. Because China is a collectivist country, many enterprises are wholly controlled by the government. Therefore, we believe that using the per-capita GDP indicator is more suitable for conducting individual-level research.

The relative economic development of areas in China is also conditioned by geographical environmental factors. For example, the overall population density in the southeast is greater than in the northwest, which may be related to the comfort provided by the local climate [60]. However, over the past 40 years, coastal areas have been substantially ahead of inland areas in terms of economic development because of their transport and other advantages conferred by reforms, a policy of opening up and other major national strategies. Both the direct effects of the climate and the indirect effects of population density and transport exert a comprehensive influence on economic development.

Differences in economic development influence the level of public social trust, but it is notable that the relationship between economic growth and social trust is more complicated than the other three relationships mentioned above. This is shown in inconsistencies between the findings of existing studies. For instance, many Western studies have suggested that economic development can have a positive influence on the social trust of local residents [61,62,63,64]. This is because a higher economic level can help individuals solve more difficulties in life and reduce their sensitivity to conflicts of social interest. As a result, they maintain a more peaceful relationship with others and a higher level of social trust.

However, studies against the cultural background of China are not consistent with these findings. A series of studies conducted by Xin and Liu [65] and Z. Xin and S. Xin [66] found that faster economic growth had a negative influence on the social trust of local residents. A higher degree of marketization led to more emphasis on profit, which enhanced the profit-seeking nature of individual decision-making, more selfish behavior, and less social trust [67]. While these findings may contradict each other, economic development is undoubtedly an important factor and, for this reason, we have given full consideration to the possible effects of this factor in our study.

### 2.3. Tourism Activities and Social Trust

#### 2.3.1. Social Exchange Theory

Social exchange theory is currently one of the most important theoretical frameworks used worldwide to interpret the relationship between tourism activities and social trust [68,69,70]. From the perspective of social exchange theory, social interaction occurs when tourists exchange information, thoughts, and other resources in a shared space with local residents or other tourists [71,72,73]. This social interaction is the foundation of social exchange, and the interaction between residents and tourists is likely to offer an opportunity for a beneficial and gratifying exchange [73,74,75,76,77,78,79].

In general, social exchange in tourism activities includes multiple forms of resource exchange: physical, social, and psychological [80,81,82]. Tourism activities allow the social exchange of these three kinds of resources between tourists and residents, which lays a key foundation for increasing their level of social trust. Research carried out by Stolle et al. suggests that social trust is developed largely through moderately intensive social contact with different individuals [12,83,84,85,86]. Compared with the daily interactions among residents, tourism activities can accelerate the development of transitional social ties. Tourism activities serve as a social platform for strangers to interact with each other. Tourists benefit from the kindness of strangers in social interactions.

These seemingly transient interactions constitute the tourist experience, and in the long run, may have a profound influence on tourists and host communities [78,79,87,88,89]. Hence, social interactions in tourism activities are more favorable for increasing the level of social trust of residents in the tourist-generating areas and destinations.

In recent years, a growing number of studies have noticed that tourism activities not only accelerate economic growth but also play a pivotal and positive role in social governance. For example, experimental research performed by Zhou [90] found that hitchhiking remarkably enhanced the level of social trust of tourists. Zhou [90] argued that the reason behind this phenomenon was that social interaction was an important part of the hitchhiker experience since they left their familiar social environment and tried to communicate with strangers. Hitchhikers could experience strong reciprocity and gratitude through social interactions with people who offered them help and showed them kindness, and so showed a higher level of social trust and willingness to engage in pro-social behavior. Research performed by Strzelecka and Okulicz-Kozaryn [91] in a large-scale social survey also fully supported the social exchange theory, finding a positive correlation between the growth of tourism in European destinations and the social trust of the residents.

#### 2.3.2. Embodied Cognition Theory

Social exchange theory relates to human interactions in tourism activities and interprets the direct effects of tourism activities on individual social trust. Embodied cognition theory, in contrast, starts from the perspective of person–land interaction in tourism activities and explains the indirect effects of tourism activities on individual social trust. Embodied cognition theory argues that the basic reason that tourism can exert a positive influence on individual social trust is that the environment can influence individual psychology and individual behavior [92,93,94]. The impact of the environment on an individual’s life shows two sides, that is, some environments may have a positive impact on an individual, while some environments may have a negative impact [95,96,97].

In terms of the positive aspects, various researchers found that more exposure to the natural environment can significantly enhance the quality of life, well-being, and mental health of individuals [98,99,100,101,102,103]. This is because the characteristics of the natural environment can have a strong impact on the positive mental state of an individual [104,105,106,107,108]. A large number of studies have revealed the physiological basis for this phenomenon in depth. Their research found that the natural environment and a pleasant sensory experience stimulate low-frequency alpha rhythms in the frontal lobe of the brain, reflecting a lower level of stress in the body and a state of relaxation and calm [103,109,110].

In terms of the negative aspects, staying in the city environment where one lives and works hinders an individual from maintaining a positive mental and physical state [111,112]. Halonen et al. [113]. and Orban et al. [114] found that industrial smells and noise around urban buildings exerted a negative influence on the emotional state and mental health of individuals. Research carried out by Lu, Lee, Gino, and Galinsky [115] also found that air pollution affected positive emotions and had a negative impact on individual well-being. Zheng, Wang, Sun, Zhang, and Kahn [116] suggested that a happy mood implied in the messages posted on social media declined significantly with the rise of PM2.5. Air pollution affects the expression of positive emotions and evokes more negative emotions, the most obvious one being anxiety. This is largely because air pollution has long been closely related to death anxiety, as the anxiety induced by air pollution resembles death anxiety [117,118].

Generally speaking, most tourism activities are based on moving from the cities or villages where people live and work to natural or cultural scenic spots that are more beautiful and comfortable. Tourists can temporarily escape the negative effects of their usual environment and also benefit from the restorative and positive effects of the natural environment. The facilitation effect of tourism activities on the emotions, well-being, quality of life, and mental health of individuals is the basis for the establishment of sound social trust.

### 2.4. Research Aims and Hypotheses

In summary, from the perspective of the relationship between the geographic environment and social trust, and the relationship between tourism activities and social trust, the long-term environment determines the mentality and behavior of people, whereas the short-term environment changes their mentality and behavior. It is notable that, on the one hand, individual behavior research is highly situational, which means cross-situational inferences cannot be drawn in relatively independent situations and specific environments. On the other hand, individual behavior is also continuous—that is, the behavioral stability of an individual will not be simply interrupted by the situation in which the individual finds themself. Given that the living environment and tourism environment are the two fundamental forms of their human–land relationship, they are connected across time and space and are part of the life-long development of an individual. They both influence an individual’s social trust. Individual behavior is characterized by both situationality and continuity. There is a complex interaction between tourism activities and the geographical environment. Therefore, it is necessary to explore the interaction mechanism between tourism activities, geographical environment, and human life [119].

Especially for China, its land area is much larger and its geographical environment is more diverse. Therefore, in the same political system and cultural environment, geography may have a more diverse influence on public psychology. In addition, tourism activities, as one of the most important large-scale spatial activities for the public, are also very popular in China. According to statistics from the National Bureau of Statistics of China, the number of domestic tourists received by each province this year reached 4.891 billion. Therefore, taking Chinese people as the research object will be more helpful in exploring how geographical environment factors and tourism activities have an interacting impact on individual psychology.

It should be noted that the impact of tourism activities includes both the impact of receiving tourists on local residents and the impact of their own travel. The former can be evaluated by the tourist reception in the destination, which means that the comprehensive statistics of local hotels, scenic spots, and transportation can roughly estimate the number of local tourists received. But the latter is often difficult to calculate. The China Tourism Academy (Data Center of the Ministry of Culture and Tourism) calculates the travel index of residents in various regions of the country every year through reverse tracing and random sampling surveys of tourist destinations. China Tourism Academy pointed out that this index is currently the only statistical basis that can relatively accurately evaluate the level of travel among residents in various regions (https://www.ctaweb.org.cn/cta/jgzz/202103/2ff33e8325264f0d88469f85f12a0dea.shtml; accessed on 19 May 2023). It is important to point out that the index is hierarchical data, not continuous data.

Based on this, this study explored whether the long-term influence of the geographical environment and the short-term influence of tourism activities would produce stable interactive effects on the level of public social trust. The influence of tourism activities on the social culture of different areas takes two specific forms—receiving tourists and supplying tourists. Our question was whether the influencing mechanism of these interactive effects on the public social trust in tourist destinations is consistent with the influencing mechanism of the interactive effects on the public social trust in tourist origin. Based on the aforesaid theoretical basis, this study proposes the following hypotheses:

**H1a.**  *Temperature and tourist reception affect the level of social trust of the people in a tourist destination.*

**H1b.**  *Temperature and tourist supply affect the level of social trust in an area from which tourists originate.*

**H2a.**  *Rice-growing and tourist reception affect the level of social trust in a tourist destination.*

**H2b.**  *Rice-growing and tourist supply affect the level of social trust in an area from which tourists originate.*

**H3a.**  *Pathogen stress and tourist reception affect the level of social trust in a tourist destination.*

**H3b.**  *Pathogen stress and tourist supply affect the level of social trust in an area from which tourists originate.*

**H4a.**  *Economic development and tourist reception affect the level of social trust in a tourist destination.*

**H4b.**  *Economic development and tourist supply affect the level of social trust in an area from which tourists originate.*

## 3. Method

### 3.1. Data Source and Sample

Data on individual respondents used in this study are from 2017 data of the China General Social Survey Database (CGSS; http://cgss.ruc.edu.cn; accessed on 3 June 2023). The database was built by the country and is the largest and highest-level social general survey database in China for now. This database was established in 2000 and is updated every 3–5 years. The data used in this study were just released in 2020, and are the latest CGSS data of 2017. The CGSS Database has been used by a great number of researches owing to its numerous merits, such as rigorous sampling and wide coverage, and has shown good results. CGSS data of 2017 used in this study included a total of 12,482 respondents, their province, gender, age, educational level, and economic income are shown in Table A1 (Appendix A).

### 3.2. Variable

#### 3.2.1. Social Trust

The variable of the level of public social trust is calculated by the two items of the social trust dimension in a CGSS scale of 2017 (http://cgss.ruc.edu.cn/info/1014/1019.htm; accessed on 3 June 2023). The CGSS project is a national academic research survey conducted with the support of special funds from the Chinese government. The CGSS scale is compiled by the China Survey and Data Center, Renmin University of China. This scale consists of 783 questions, which cover a large number of research contents such as individual basic demographic variables and social psychological variables. The CGSS annual data used in the current study was collected by 40 universities across the country, and the whole research process took seven months. CGSS is open for free use by all social science researchers in China. As of the latest statistics, CGSS data has supported 2470 research publications, including 355 papers in international English journals (http://cgss.ruc.edu.cn/info/1014/1018.htm; accessed on 12 June 2023). The current study uses data from the Social Trust Scale. The scale consists of two items: (1) In general, do you agree that the vast majority of people in this society are trustworthy? (2) In general, do you agree that other people in this society will try to take advantage of you if you are careless? Item 2 is a reverse score question. The total score of these two questions represents the level of social trust of the respondents [24,25].

#### 3.2.2. Temperature

The variable of temperature is based on the calculation model of temperature data adopted in a study by Vliert [39]. This study first collected the annual average temperature of different provinces in China from 1996 to 2017 from the Yearbook Database of the National Bureau of Statistics of China (http://www.stats.gov.cn/tjsj/ndsj/; accessed on 12 June 2023) and then calculated the difference between the annual average temperature of different provinces and 22°—the temperature most suitable for humans to live (see Table A2). The absolute value of the difference represents the degree to which the temperature throughout the year is suitable for local residents to live. The smaller the absolute value, the higher the temperature suitability, and vice versa.

#### 3.2.3. Rice-Growing Areas

**Figure 1:**
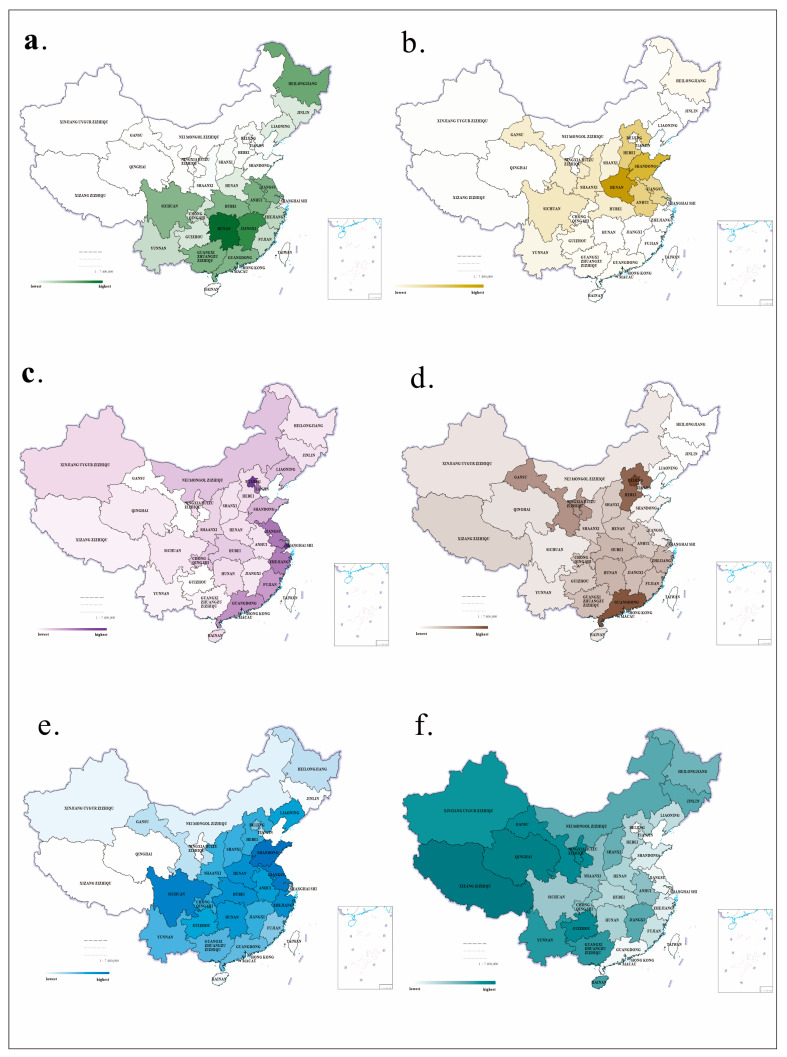
Comparative trend chart of the variables in the study by province. (**a**) Comparative trend chart of rice planting area in each province from 1996 to 2017. Unit: thousands of hectares. (**b**) Comparative trend chart of wheat planting area in each province from 1996 to 2017. Unit: thousands of hectares. (**c**) Comparative trend chart of Per capita GDP in each province from 1996 to 2017. Unit: yuan. (**d**) Comparative trend chart of the number and incidence of influenza in each province from 2004 to 2017. Unit: thousands of hectares. (**e**) Comparative trend chart of the number of domestic tourists received by each province from 2013 to 2017. Unit: 100 million person-times. (**f**) Comparative trend chart of travel index of each province from 2013 to 2017.

The variable of rice-growing is based on the encoding model used in research carried out by Talhelm et al. [50] This study first collected data on rice-, wheat-, and corn-growing areas in Chinese provinces from 1996 to 2017 from the Yearbook Database of the National Bureau of Statistics of China (http://www.stats.gov.cn/tjsj/ndsj/; accessed on 12 June 2023), and then obtained the rice-growing ratios of different places by calculating the rice-growing area/(wheat-growing area + corn growing area; see Table A3, Table A4 and Table A5; see the trend chart in Figure 1). According to the grouping method used by Talhelm et al. [50], the area where the growing ratio was higher than 1 was the rice-growing area, whereas the area where the growing ratio was lower than 1 was the wheat-growing area.

#### 3.2.4. Pathogen Stress

The variable of pathogen stress is based on the prevalence of influenza in various areas. As the most typical and common infectious disease in the world, influenza extensively affects the daily lives of people. Hence, this study collected data on the prevalence of influenza in different provinces from 2004 to 2017 from the National Public Health Science Database of the China Population and Health Scientific Data Sharing Platform (https://www.phsciencedata.cn/Share/index.jsp; accessed on 17 June 2023; see Table A6; see Figure 1). The averages of these data reflect the chronic pathogen stress of people in different places.

#### 3.2.5. Economic Development Level

The economic development level is measured through data on traditional per-capita GDP. The data are also from data on the average per-capita GDP of different provinces in China from 1996 to 2017 recorded in the Yearbook Database of the National Bureau of Statistics of China (http://www.stats.gov.cn/tjsj/ndsj/; accessed on 17 June 2023; see Table A7; see Figure 1). These figures reflect the differences between different places in the level of economic development.

#### 3.2.6. Level of Tourist Reception

According to the statistical data from the China Tourism Yearbook (see Table A8; see Figure 1), this study uses the average numbers of tourists received by various provinces from 2013 to 2017 to signify the tourist reception level of each province.

#### 3.2.7. Level of Tourist Supply

The level of tourist supply is denoted by the travel index of the residents of each province, which is calculated through annual big data related to tourism of the same year from the China National Tourism Administration (see Table A9; see Figure 1). The higher the index, the more trips are taken by the residents in this province. The variable uses the average data from 2013 to 2017 as the indicator of the level of tourist supply in each province.

Data on social trust as a dependent variable are from the 2017 CGSS Database. Data on temperature, rice-growing, and economic development as independent variables are the averages from 1996 to 2017, pathogen stress data are from 2004 to 2017. The reason for this is that we aimed to discuss the long-term effects of the living environment on the mentality of people on the one hand, but standardized and authoritative data exist only from 1996 and 2004, respectively, when China established a methodical National Statistical Yearbook System and National Public Health Science Database. Different from the aforesaid independent variables, tourism activities exert short- and medium-term effects rather than the long-term effects of residential activities. Therefore, this study used the averages over more recent years (2013 to 2017) as the indicators of tourist reception level and tourist supply level.

## 4. Results

All statistical analyses in this study were carried out with the use of SPSS 26.0. The dependent variable of public social trust is significantly positively correlated with the level of tourist reception, significantly negatively correlated with the level of pathogen stress, and not significantly correlated with other independent variables. In addition, the specific relationships between other independent variables are shown in Table 1.

**Figure 2:**
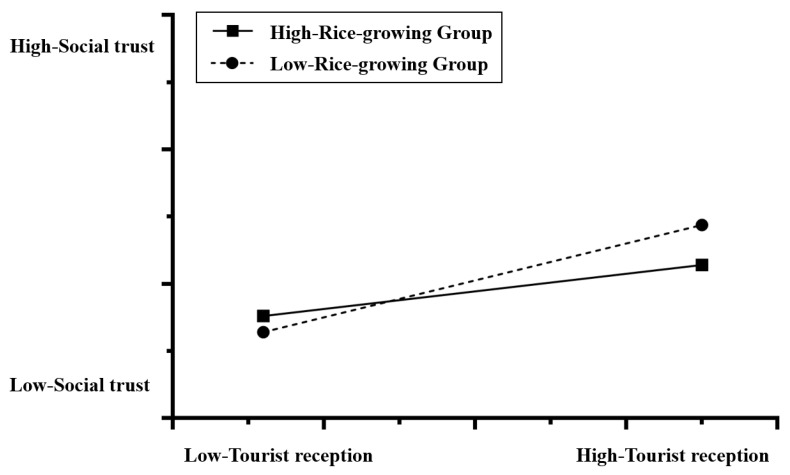
The interaction between tourist reception and rice-growing on social trust.

**Figure 3:**
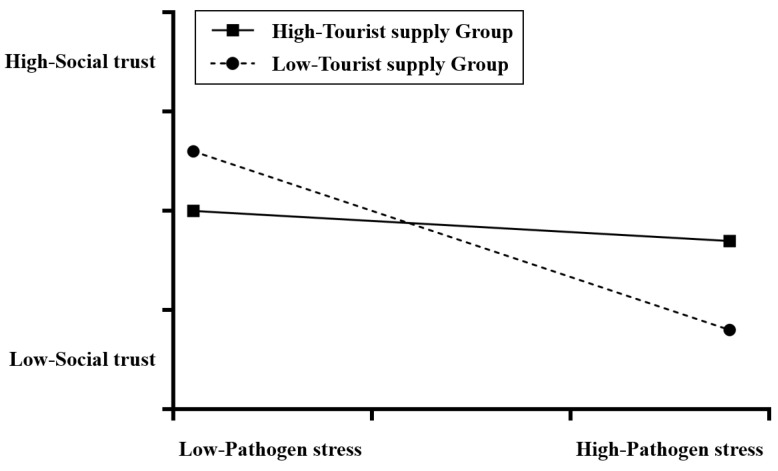
The interaction between pathogen stress and tourist supply on social trust.

**Figure 4:**
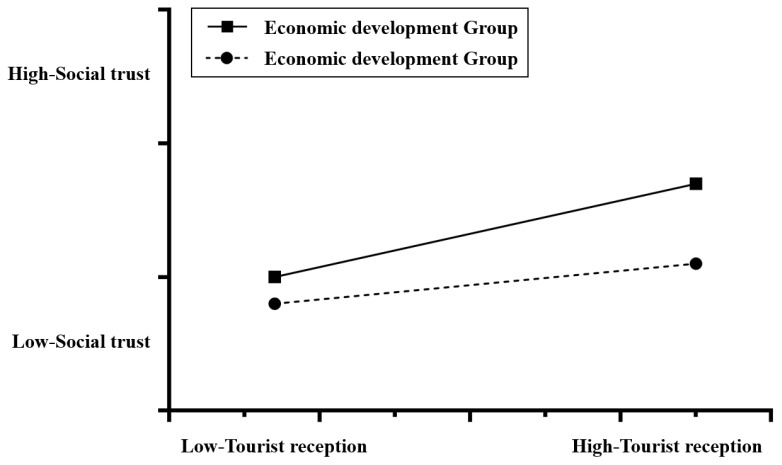
The interaction between tourist reception and economic development on social trust.

The results of current hypothesis testing (Table 2) show that the interaction between the level of tourist reception and temperature is insignificant, and the interaction between the level of tourist supply and temperature is also insignificant. This result does not support hypotheses H1a and H1b. In other hypothesis tests, the interaction between the level of tourist reception and the level of rice-growing, the interaction between the level of tourist supply and pathogen stress, and the interaction between the level of tourist reception and the level of economic development are significant. These results support H2a, H3b, and H4a. However, H2b, H3a, and H4b, are not supported by the data. Figure 2 shows that in the interaction between the level of tourist supply and the level of rice-growing, the upward trend of rice-growing areas is significantly greater than that for wheat- and corn-growing areas; in the interaction between the level of tourist reception and pathogen stress (Figure 3), the slope of the group with a larger number of tourists is lower than that of the group with a smaller number of tourists; and in the interaction between the level of tourist reception and the level of economic development (Figure 4), the slope of the low per-capita GDP group is significantly higher than that of the high per-capita GDP group.

## 5. Discussion

Our analysis shows that public social trust is significantly correlated with the level of local tourist reception but is not directly connected with the level of tourist supply. For tourism activities, receiving and supplying tourists have different effects on local social culture. Receiving tourists has a direct effect with the larger the number of tourists received in an area, the higher the level of public social trust. Although supplying tourists does not have direct effects on the level of social trust of the local residents, it may have indirect effects by moderating the intensity of the influence of other factors on social trust. Therefore, this study also concentrates on testing and comparing how the level of social trust is influenced by residents in destinations and tourist-generating areas, respectively. We did not find a significant correlation between temperature and the level of social trust of local residents. This is inconsistent with previous findings [34,35,38,45,46]. We also found that there was no interaction between temperature and the level of tourist reception or the level of tourist supply. This result indicates that temperature does not exert any direct influence on public social trust nor has an indirect influence on it together with tourism activities. In our opinion, the inconsistency between findings based on different cultures may be caused by regional factors. A major difference between this study and previous studies is that we explored the effects on Chinese people. As one of the few socialist countries in the world, China is different from other countries in many ways. Since the founding of New China, the country has developed a residential guarantee strategy of unified heat supply for 4–6 months every year in most areas north of the Qinling Mountains, and supplies heat or stops it according to the specific temperature changes in different provinces each year. Subsidized by central financing, the heating fee for each household is only 5.6 yuan/square meter/month. As a result, the cost of heating is not an economic burden and temperature-induced survival pressures do not exist. Therefore, temperature, has no further influence on the social trust of the local residents, nor will it further interact with tourism activities to have an impact.

Through testing the interaction between rice-growing and tourist reception level or tourist supply level, we found that, on one hand, rice-growing did not show a significant correlation with the social trust level of local residents. On the other hand, we found that the rice-growing ratio and the level of tourist reception have interacting effects on the social trust of the local residents. Although an increase in the number of tourists received helps boost the social trust of local residents, this influence is not consistent in all parts of the country, as it is moderated by the farming culture stressed in the rice theory. Specifically, the abovementioned interacting effects are that for the people in the wheat and corn-growing areas with a low rice-growing ratio, tourist reception activities can boost the level of public social trust more significantly. This influence is weak in rice-growing areas with a high rice-growing ratio. In other words, for the same level of tourist reception activities, its effect of improving social trust in wheat-growing areas is markedly better than that in rice-growing areas. The possible reason behind this phenomenon is that, according to the rice theory, the rice-growing culture itself shapes a social culture of closer local community ties, whereas the wheat-growing culture gives rise to weak distant- and extra-community relations. Therefore, compared with the positive effects of the rice-growing culture, the effects of wheat-growing activities on the society, culture, and mentality of the local residents are slightly negative and have greater potential for improvement. This finding is to some extent consistent with previous studies [50,51,52,53,54,55,56]. We further found that, on the basis of traditional rice theory, this study revealed two new social phenomena: first, the sociocultural impact of rice-growing activities has a direct and independent impact (as found in previous studies), as well as an indirect impact. Our study found that the rice-growing ratio moderates the influence of the level of tourist reception on the social trust of the local residents; second, the rice-growing ratio has interacting effects only with the level of tourist reception and has no interacting effect with the level of tourist supply.

Our findings on the influence of pathogen stress and tourism activities on public social trust found a close relationship between the level of pathogen stress and the social trust level of local residents. This result is completely consistent with the prediction of the pathogen stress theory [58,59], in that the incidence of epidemics in the place of residence not only influences the health of people, but also influences the level of social trust. In addition, we found that pathogen stress can directly influence public social trust as well as exert interacting effects on the level of tourist supply. Although pathogen stress has a negative influence on public social trust, this influence varies in different areas. This negative influence is weaker in areas with a higher level of tourist supply and is stronger in areas with a lower level of tourist supply. This shows that tourism activities can mitigate the negative influence caused by pathogen stress. It is worth mentioning that, at present, this influence is only found in tourist-generating areas, not in destinations. This also suggests that transporting tourists and receiving tourists have different influences on local society and culture.

We also found that there are interacting effects of economic development level and tourism activities on public social trust. We did not find a direct connection between the level of local economic development and the level of public social trust in the correlation analysis. However, we found that the level of economic development moderated the influence of the level of tourist reception on the social trust of local residents. In other words, it does not come into play independently but rather interacts with tourism activities to influence public social trust. This interaction is manifested in the fact that in more economically developed areas with lower per-capita GDP, tourist reception activities had a greater influence on improving the social trust level of the local residents. These positive effects also existed in economically developed areas with higher per-capita GDP, but the level was lower. Irrespective of the level of economic development of an area, tourist reception activities can exert a high level of positive effects on local society and culture. A more important finding of this study was that, for less economically developed areas, actively developing tourism is a strategy that brings double benefits. It stimulates the growth of the local economy and facilitates the improvement of social trust.

### 5.1. Theoretical Contribution

This study brings a new way of thinking to theories in the field in that it explores the interacting effects of geographical environmental factors in residential activities and tourism activities on public social trust. It reveals the differentiated influence of these interacting effects on the social trust of residents in destinations and tourist-generating areas in more depth. China covers a vast territory with a large population. The findings of this study have added value to previous studies on the relationships between geographical environment, tourism activities, and public social trust, and provided cultural research evidence from China. The study has also, for the first time, revealed interacting effect mechanisms on public social trust from the perspective of the cross-over study of geographical environment and tourism activities. In view of the multifold advantages of this study, including wide sampling distribution, highly rigorous sampling, and the reasoned choice of variables and calculation methods, the findings are highly representative and valuable. In addition, the findings of this study have certain social and practical significance. In the current era, global epidemics are frequent, which can have a negative impact on interactions between cities. Therefore, it is important for countries around the world to recover tourism after every pandemic. This significance is not limited to the contribution of tourism to the national economy, but more importantly, to the direct and indirect enhancement of public social trust. Helping the public rebuild social trust does more good for the recovery of the market economy, for accelerating the restoration of normal social order, and for exerting a more extensive, positive, and persistent influence. In brief, this study hopes to urge the country to attach more importance to tourism development and research in the field of tourism through the abovementioned findings, thus helping tourism research contribute to the improvement of national social governance in a more effective way.

### 5.2. Limitations and Future Research Directions

Although current research focuses on promoting the development of existing theories, it still has the following limitations: (1) the current research is still a variable-centered research rather than an individual-centered research. Therefore, there is a lack of exploration of deeper individual-level characteristics; (2) the samples for the current study were all from China. Although to a certain extent, this avoids the influence of political system and cultural background, the current conclusions also lack a cross-cultural adaptability test; (3) the current study lacks evidence of long-term longitudinal tracking data. Although it is difficult to carry out, longitudinal research plays an important role in delving deeper into the interacting impact mechanism of geographical environment and tourism activities on public social trust. We believe that this is also an urgent need to be carried out in the future.

## 6. Conclusions

The main conclusion drawn by this study includes the following aspects: (1) the direct effect of pathogen stress and tourism activities on public social trust is much higher than that of temperature, rice-growing, and economic development; (2) geographical environment and tourism activities have interacting effects on public social trust. This influencing mechanism is specifically manifested as rice-growing and tourist reception levels can have interacting effects on the social trust of the residents in a tourist destination. That is to say, for the people in the wheat- and corn-growing areas with a low rice-growing ratio, tourist reception activities can boost the level of public social trust more significantly; (3) pathogen stress and tourist supply level can exert interacting effects on the social trust of the residents in an area from which tourists originate. In areas with a higher level of tourist supply, this negative influence of pathogen stress is weaker; (4) economic development and tourist reception can have interacting effects on the social trust of the residents in a tourist destination. This interaction is that in more economically developed areas with lower per-capita GDP, tourist reception activities had a greater influence on improving the public social trust.

## Figures and Tables

**Table 1:** Descriptive statistics and correlations.

Item	M	SD	ST	TR	TS	TE	RG	PS	ED
ST	6.395	1.661	-						
TR	3.694	1.698	0.020 *	-					
TS	11.929	8.112	−0.004	−0.113 **	-				
TE	6.899	4.194	−0.009	−0.418 **	0.267 **	-			
RG	1.376	0.484	0.010	−0.048 **	−0.187 **	−0.673 **	-		
PS	11.081	7.617	−0.029 **	−0.158 **	−0.418 **	−0.322 **	0.198 **	-	
ED	30,088.973	16,983.457	−0.009	−0.302 **	−0.825 **	−0.131 **	0.180 **	0.494 **	-

Note: ST = Social trust; TR = Tourist reception; TS = Tourist supply; TE = Temperature; RG = Rice-growing; PS = Pathogen stress; ED = Economic development; * *p* < 0.05; ** *p* < 0.01.

**Table 2:** Hierarchical regression analyses predicting generalized trust.

Model	B	SE	β	R^2^	ΔR^2^	F	t
Model 1a-1							
TR	0.0189	0.0096	0.0194	0.0001	0.0001	2.458	1.9649 *
TE	0.0004	0.0039	0.0011				−0.1125
Model 1a-2							
TR	−0.0097	0.0225	−0.0099	0.001	0.0001	2.301	−0.4319
TE	−0.0098	0.0077	−0.0247				−1.2721
TR × TE	0.0038	0.0027	0.0316				1.4096
Model 1b-1							
TS	0.0004	0.0019	−0.0017	0.0001	0.0001	0.544	−0.1846
TE	−0.0035	0.0037	−0.0087				−0.9400
Model 1b-2							
TS	0.0001	0.0037	0.0001				0.0059
TE	−0.0026	0.0085	−0.0065	0.0001	0.0001	0.367	−0.3006
TS × TE	−0.0001	0.0005	−0.0034				−0.1162
Model 2a-1							
TR	0.0206	0.0089	0.0208	0.001	0.0001	3.337 *	2.3157 *
RG	0.0386	0.0308	0.0113				1.2543
Model 2a-2							
TR	0.1082	0.0270	0.1093				4.0032 ***
RG	0.2925	0.0801	0.0854	0.001	0.001	6.152 **	3.6505 ***
TR × RG	−0.0693	0.0202	−0.1168				−3.4318 ***
Model 2b-1							
TS	0.0005	0.0019	−0.0024	0.0001	0.0001	0.689	−0.2586
RG	0.0337	0.0313	0.0098				1.0763
Model 2b-2							
TS	−0.0005	0.0057	−0.0024				−0.0886
RG	0.0336	0.0538	0.0098	0.0001	0.0001	0.459	0.6243
TS × RG	0.0001	0.0040	0.0001				0.0026
Model 3a-1							
TR	0.0153	0.0089	0.0156	0.001	0.001	6.738 **	1.7261
PS	−0.0058	0.0020	−0.0265				−2.9274 *
Model 3a-2							
TR	0.0325	0.0154	0.0333				2.1072 *
PS	0.0016	0.0058	0.0075	0.001	0.001	5.112 **	0.2825
TR × PS	−0.0025	0.0018	−0.0378				−1.3638
Model 3b-1							
TS	−0.0040	0.0020	−0.0196	0.001	0.001	7.225 **	−1.9881 **
PS	0-.0081	0.0021	−0.0372				−3.7743 ***
Model 3b-2							
TS	−0.0214	0.0037	−0.1047				−5.8522 ***
PS	−0.0193	0.0029	−0.0886	0.004	0.004	15.644 ***	−6.6366 ***
TS × PS	0.0016	0.0003	0.0939				5.6962 ***
Model 4a-1							
TR	0.0185	0.0092	0.0190	0.0001	0.0001	2.496	2.0210 *
ED	−0.0001	0.0001	−0.0028				−0.2987
Model 4a-2							
TR	0.0851	0.0214	0.0871				3.9689 ***
ED	0.0001	0.0001	0.0593	0.001	0.001	5.597 **	2.9101 **
TR × ED	−0.0001	0.0001	−0.0830				−3.4342 ***
Model 4b-1							
TS	−0.0071	0.0032	−0.0348	0.0001	0.0001	2.869	−2.1949 *
ED	−0.0001	0.0001	−0.0373				−2.3496 *
Model 4b-2							
TS	−0.0075	0.0051	−0.0366				−1.4560
ED	−0.0001	0.0001	−0.0371	0.0001	0.0001	1.911	−2.3242 *
TS × ED	0.0001	0.0001	0.0021				0.0929

Note: TR = Tourist reception; TS = Tourist supply; TE = Temperature; RG = Rice-growing; PS = Pathogen stress; ED = Economic development; * *p* < 0.05; ** *p* < 0.01; *** *p* < 0.001.

## Data Availability

The original contributions presented in the study are included in the article, further inquiries can be directed to the corresponding authors.

## Appendix A

Appendix A in this study includes Table A1, Table A2, Table A3, Table A4, Table A5, Table A6, Table A7, Table A8 and Table A9.

**Table A1:** Demographic information of participants.

Item	Category	Frequency	Percentage
	Shanghai	991	7.9%
	Yunnan	418	3.3%
	Beijing	1082	8.7%
	Jilin	504	4.0%
	Sichuan	606	4.9%
	Tianjin	400	3.2%
	Ningxia	100	0.8%
	Anhui	418	3.3%
	Shandong	600	4.8%
	Shanxi	303	2.4%
	Guangdong	575	4.6%
	Guangxi	398	3.2%
	Jiangsu	498	4.0%
Province	Jiangxi	503	4.0%
	Hebei	295	2.4%
	Henan	600	4.8%
	Zhejiang	496	4.0%
	Hubei	605	4.8%
	Hunan	501	4.0%
	Gansu	200	1.6%
	Fujian	299	2.4%
	Guizhou	300	2.4%
	Liaoning	406	3.3%
	Chongqing	300	2.4%
	Shanxi	397	3.2%
	Qinghai	100	0.8%
	Heilongjiang	587	4.7%
Gender	Male	5894	47.2%
	Female	6588	52.8%
	15–24	810	6.5%
	25–34	1719	13.8%
	35–44	1901	15.2%
Age (years)	45–54	2656	21.3%
	55–64	2437	19.5%
	65–74	1917	15.4%
	75–84	875	7%
	85 and above	167	1.3%
	No education	1510	12.1%
	Private schools and literacy classes	91	0.7%
	Primary school	2686	21.5%
	Junior middle school	3483	27.9%
	Vocational high school	163	1.3%
	Ordinary high school	1468	11.8%
Educational level	Secondary specialized school	539	4.3%
	Technical school	72	0.6%
	College (adult higher education)	378	3.0%
	College (formal higher education)	672	5.4%
	Undergraduate (adult higher education)	299	2.4%
	Undergraduate (formal higher education)	948	7.6%
	Graduate and above	173	1.4%
	10,000 and below	4686	37.5%
	10,001–20,000	1424	11.4%
	20,001–40,000	3265	26.2%
	40,001–60,000	1589	12.7%
	60,001–80,000	492	3.9%
Income (yuan)	80,001–100,000	470	3.8%
	100,001–120,000	114	0.9%
	120,001–140,000	28	0.2%
	140,001–160,000	102	0.8%
	160,001–180,000	19	0.2%
	180,001–200,000	119	1%
	200,001 and above	174	1.4%

**Table A2:** The average temperature of each province from 1996 to 2017 (unit: °C).

Province	2017	2016	2015	2014	2013	2012	2011	2010	2009	2008	2007
Beijing	14.2	13.8	13.7	14.1	12.8	12.9	13.4	12.6	13.3	13.4	14.0
Tianjin	14.2	13.8	13.7	14.0	12.8	12.5	12.9	12.2	12.9	13.3	13.6
Hebei	15.0	14.6	14.6	14.9	13.8	14.0	14.2	14.0	14.4	14.6	14.9
Shanxi	11.5	11.2	11.3	10.9	11.2	10.7	10.8	11.3	11.1	10.9	11.4
Liaoning	9.3	8.8	9.0	9.2	7.9	7.4	7.7	7.2	7.7	8.6	9.0
Jilin	7.0	6.6	7.2	7.1	5.6	5.2	5.9	5.2	6.1	7.2	7.7
Heilongjiang	5.1	5.0	5.6	5.1	4.3	4.6	5.2	4.5	5.0	6.6	6.7
Shanghai	17.7	17.6	17.0	17.0	17.6	16.9	16.9	17.2	17.4	17.2	18.2
Jiangsu	17.0	16.8	16.4	16.4	16.8	16.0	16.1	16.2	16.4	16.1	17.3
Zhejiang	18.3	18.2	17.5	17.5	18.0	17.1	17.2	17.4	17.8	17.5	18.4
Anhui	17.1	17.0	16.7	16.5	17.0	16.5	16.3	16.4	16.7	16.4	17.3
Fujian	21.2	21.0	20.7	20.8	20.4	20.2	20.2	20.4	20.7	20.4	21.0
Jiangxi	19.2	19.0	18.7	18.8	19.0	18.0	18.4	18.5	18.8	18.5	19.2
Shandong	15.7	15.4	15.0	15.4	14.7	14.3	14.1	14.3	14.8	14.6	15.0
Henan	16.8	16.4	15.9	16.3	16.1	15.5	15.1	15.6	15.5	15.6	15.9
Hubei	17.3	17.3	16.8	16.7	17.1	16.4	16.3	16.6	17.9	17.6	18.5
Hunan	17.7	17.5	17.4	18.6	19.2	17.6	17.9	18.2	18.5	18.3	18.8
Guangdong	22.1	21.9	22.3	21.7	21.5	21.7	21.4	22.5	23.0	22.4	23.2
Guangxi	21.9	22.3	22.2	21.6	21.6	21.4	20.7	21.8	22.2	20.8	21.7
Chongqing	19.4	19.5	19.6	18.6	19.8	18.3	18.8	18.6	19.0	18.5	19.0
Sichuan	16.6	16.8	16.8	16.0	16.9	15.9	15.9	16.0	16.8	16.3	16.8
Guizhou	15.2	15.3	15.2	14.7	15.1	13.7	14.0	14.6	14.9	14.1	14.9
Yunnan	15.7	15.8	16.2	16.4	16.0	16.3	15.5	16.7	16.6	15.4	15.6
Shaanxi	15.6	15.8	15.2	15.2	15.8	14.2	14.1	14.6	15.1	14.9	15.6
Gansu	8.0	8.2	8.3	7.7	8.3	7.5	7.7	7.9	8.0	10.6	11.1
Qinghai	6.3	6.6	6.4	5.7	6.1	5.2	5.7	6.4	6.2	5.7	6.1
Ningxia	11.0	10.7	10.7	10.7	11.2	9.8	9.9	10.3	10.5	9.9	10.4
**Province**	**2006**	**2005**	**2004**	**2003**	**2002**	**2001**	**2000**	**1999**	**1998**	**1997**	**1996**
Beijing	13.4	13.2	13.5	12.8	13.1	12.9	12.8	13.1	13.1	13.1	12.7
Tianjin	13.2	12.9	13.2	12.7	13.2	13.0	12.9	13.1	13.4	13.1	12.2
Hebei	14.6	14.3	14.3	13.6	14.4	14.4	13.9	14.7	15.0	14.4	13.5
Shanxi	11.8	10.9	10.9	10.1	10.9	11.0	10.7	11.5	11.5	10.1	9.8
Liaoning	8.3	8.0	9.6	9.0	9.2	8.4	8.3	8.9	9.7	8.8	8.1
Jilin	6.6	5.6	7.1	7.0	6.8	6.1	5.6	6.0	7.4	6.7	5.9
Heilongjiang	5.3	4.7	5.8	5.9	5.4	4.8	4.6	4.8	5.5	5.7	5.0
Shanghai	17.9	17.1	17.5	17.0	17.5	17.2	17.2	16.6	17.8	16.9	16.2
Jiangsu	16.9	16.3	16.9	16.0	16.6	16.6	16.4	15.7	16.7	16.2	15.4
Zhejiang	18.2	17.5	17.8	17.4	17.4	17.3	17.2	16.7	17.9	17.1	16.5
Anhui	17.0	16.2	16.6	16.3	17.2	16.8	16.7	16.3	17.1	16.7	15.8
Fujian	20.8	20.3	20.8	20.9	20.9	20.6	20.5	20.4	21.1	20.1	19.9
Jiangxi	18.6	18.2	18.8	18.5	18.3	18.2	17.9	18.1	18.8	17.8	17.6
Shandong	15.3	14.4	14.8	13.8	15.0	14.6	14.5	15.1	16.0	15.4	14.7
Henan	15.8	14.9	15.5	14.4	15.4	15.1	15.0	15.4	15.5	14.9	14.2
Hubei	18.3	17.8	18.3	17.4	17.9	18.0	17.7	17.5	18.2	17.5	16.8
Hunan	18.5	17.7	18.3	17.6	17.7	17.6	17.1	17.2	18.1	17.2	16.8
Guangdong	23.2	22.8	22.8	22.9	22.9	22.5	22.5	22.4	22.8	22.0	21.6
Guangxi	22.0	21.4	21.5	22.0	21.7	21.3	21.5	21.7	23.0	22.2	21.7
Chongqing	19.2	18.6	18.4	18.8	18.7	18.8	18.2	18.4	19.2	18.5	17.7
Sichuan	16.9	16.2	16.2	17.2	17.4	17.3	16.6	16.7	17.4	16.8	16.0
Guizhou	14.8	14.1	14.6	14.8	14.6	14.5	13.8	15.9	17.3	15.4	15.0
Yunnan	16.4	16.7	15.6	16.4	16.1	16.0	15.6	16.3	16.5	15.4	15.6
Shaanxi	15.2	15.0	15.4	14.3	15.4	15.0	14.5	15.0	15.0	14.8	13.7
Gansu	8.5	7.2	10.9	10.8	11.0	11.0	11.0	11.1	11.4	10.9	9.6
Qinghai	6.4	5.8	5.8	6.0	6.1	6.0	5.8	6.1	6.3	5.5	4.9
Ningxia	10.9	10.1	10.3	9.7	10.0	10.1	9.6	9.5	10.5	10.2	9.6

**Table A3:** Rice planting area in each province from 1996 to 2017 (unit: thousands of hectares).

Province	2017	2016	2015	2014	2013	2012	2011	2010	2009	2008	2007
Beijing	0.1	0.2	0.2	0.2	0.2	0.2	0.2	0.3	0.4	0.4	0.5
Tianjin	30.5	26.5	22.2	22.3	22.3	18.6	17.4	17.9	18.1	16.3	14.9
Hebei	75.0	76.3	79.9	80.5	82.9	82.6	80.3	77.6	83.4	80.5	84.0
Shanxi	0.8	0.8	0.8	1.0	1.1	1.1	1.1	1.1	1.2	1.2	1.5
Liaoning	492.7	476.4	469.2	492.1	577.9	599.0	607.0	633.9	624.8	637.2	649.7
Jilin	820.8	800.2	778.8	757.0	739.4	711.6	697.7	680.2	667.6	665.5	671.6
Heilongjiang	3948.9	3925.3	3918.4	3968.5	3860.8	3630.7	3437.3	3139.4	2695.4	2629.2	2287.8
Shanghai	104.1	106.3	110.2	111.3	114.8	117.6	118.6	120.5	120.5	115.0	115.0
Jiangsu	2237.7	2256.3	2250.3	2236.7	2229.9	2228.9	2227.7	2224.9	2223.8	2222.6	2220.8
Zhejiang	620.7	613.1	634.2	654.2	677.1	700.1	774.5	822.5	860.8	884.9	927.1
Anhui	2605.2	2537.4	2476.4	2422.0	2320.9	2333.6	2333.9	2338.6	2356.5	2254.2	2205.6
Fujian	628.6	630.9	659.9	686.4	711.5	734.7	765.5	789.6	814.6	827.7	851.6
Jiangxi	3504.7	3527.1	3541.3	3522.6	3501.9	3476.5	3441.3	3410.4	3344.2	3313.1	3245.6
Shandong	108.9	106.7	117.2	123.2	123.9	124.5	125.1	128.7	135.0	130.9	130.6
Henan	615.0	614.1	616.4	614.7	611.0	621.8	616.3	610.8	598.7	596.4	595.9
Hubei	2368.1	2358.7	2383.4	2201.8	2202.6	2086.4	2081.1	2087.8	2093.6	1956.9	2027.2
Hunan	4238.7	4277.6	4287.8	4275.0	4218.5	4209.6	4160.8	4105.3	4103.4	3968.3	3915.2
Guangdong	1805.4	1806.0	1804.8	1826.8	1850.0	1898.2	1898.0	1918.1	1933.6	1930.7	1930.4
Guangxi	1801.7	1836.7	1871.4	1923.8	1955.8	1979.0	2012.2	2040.8	2084.0	2091.9	2112.9
Chongqing	658.9	660.9	647.1	650.8	652.4	654.8	656.8	658.1	661.2	658.9	644.3
Sichuan	1874.9	1874.0	1878.7	1892.4	1905.4	1929.8	1943.2	1966.9	1990.9	2011.6	2024.0
Guizhou	700.5	714.3	711.1	714.1	712.6	707.0	701.4	712.0	710.4	699.2	680.1
Yunnan	870.6	881.4	909.3	942.2	979.7	943.9	966.5	933.1	978.5	977.0	969.9
Shaanxi	105.6	107.4	107.5	108.7	114.3	113.9	113.1	115.3	121.8	121.4	113.8
Gansu	4.0	4.2	4.1	4.7	4.9	5.2	5.3	5.6	5.5	5.4	5.2
Qinghai	0.0	0.0	0.0	0.0	0.0	0.0	0.0	0.0	0.0	0.0	0.0
Ningxia	81.1	80.9	74.3	78.1	82.1	84.3	83.9	83.2	78.3	80.3	77.0
**Province**	**2006**	**2005**	**2004**	**2003**	**2002**	**2001**	**2000**	**1999**	**1998**	**1997**	**1996**
Beijing	0.7	0.8	0.8	1.6	4.5	6.8	14.1	19.2	19.4	23.2	23.1
Tianjin	14.1	16.7	13.7	7.0	14.9	11.4	35.4	61.1	54.4	66.4	61.7
Hebei	88.7	87.7	83.5	75.6	111.0	94.1	143.9	154.7	153.2	155.3	141.8
Shanxi	1.5	2.7	2.6	3.1	3.5	5.1	4.5	5.9	6.1	6.1	5.8
Liaoning	624.9	568.4	544.2	500.6	556.4	515.5	489.7	501.5	496.0	491.7	478.1
Jilin	656.3	654.0	600.1	541.0	666.1	686.9	584.8	465.2	459.0	453.1	434.1
Heilongjiang	1992.2	1650.3	1587.8	1290.9	1564.4	1567.0	1605.9	1614.9	1566.7	1396.9	1107.5
Shanghai	110.6	112.7	111.8	106.2	133.1	153.9	176.1	200.8	203.3	208.4	210.5
Jiangsu	2216.0	2209.3	2112.9	1840.9	1982.1	2010.3	2203.5	2398.5	2369.7	2377.6	2335.9
Zhejiang	994.5	1028.5	1028.1	979.4	1172.3	1340.0	1598.0	1940.4	2007.9	2085.9	2138.2
Anhui	2207.7	2149.1	2129.7	1972.4	2044.1	1950.1	2236.7	2145.5	2158.3	2212.1	2238.5
Fujian	890.6	951.6	985.1	962.6	1082.9	1156.5	1222.3	1373.2	1387.9	1401.6	1405.2
Jiangxi	3239.3	3129.0	3029.7	2685.3	2786.6	2808.3	2832.0	3050.0	2900.8	3063.5	3052.6
Shandong	127.3	119.8	124.4	112.6	155.3	173.6	176.8	195.8	157.6	164.7	151.6
Henan	571.3	511.1	508.5	503.0	469.4	415.9	459.6	508.5	498.4	489.5	479.9
Hubei	1975.1	2077.4	1989.6	1805.1	1932.0	1987.9	1995.3	2285.0	2239.3	2466.0	2448.6
Hunan	3931.7	3795.2	3716.8	3410.0	3541.5	3691.6	3896.1	3984.5	3976.4	4075.8	4064.1
Guangdong	1941.9	2137.6	2139.0	2130.6	2195.5	2369.3	2467.4	2557.5	2686.0	2704.1	2713.4
Guangxi	2238.1	2360.4	2356.0	2356.3	2412.6	2423.6	2301.6	2388.7	2433.5	2434.3	2430.8
Chongqing	672.3	748.0	749.3	750.5	755.2	764.0	776.6	788.6	794.7	803.9	795.7
Sichuan	2081.9	2087.5	2063.8	2040.3	2076.1	2093.1	2123.8	2176.0	2167.4	2196.1	3020.1
Guizhou	679.6	721.7	716.5	720.5	734.6	750.0	750.5	748.0	746.8	742.9	741.3
Yunnan	1029.7	1049.3	1086.2	1043.1	1083.0	1100.3	1073.6	903.0	919.6	921.2	939.2
Shaanxi	120.9	147.1	145.8	139.5	130.5	140.8	144.8	154.6	160.0	153.9	156.9
Gansu	5.3	5.1	4.9	4.8	6.3	7.1	7.2	7.0	8.4	6.8	6.7
Qinghai	0.0	0.0	0.0	0.0	0.0	0.0	0.0	0.0	0.0	0.0	0.0
Ningxia	88.3	71.3	64.4	46.7	76.4	74.2	76.7	71.0	66.5	67.2	64.0

**Table A4:** Wheat planting area in each province from 1996 to 2017 (unit: thousands of hectares).

Province	2017	2016	2015	2014	2013	2012	2011	2010	2009	2008	2007
Beijing	11.3	15.9	20.8	23.6	36.2	52.2	58.1	61.6	60.6	63.9	41.4
Tianjin	108.8	107.3	106.0	108.0	107.8	110.9	110.4	109.3	109.1	107.0	104.5
Hebei	2373.4	2389.8	2394.2	2404.0	2432.0	2457.1	2435.0	2451.4	2397.8	2431.8	2420.2
Shanxi	560.5	564.0	575.9	585.1	598.7	619.7	650.1	678.8	689.9	673.2	699.8
Liaoning	3.6	2.9	3.0	3.3	3.5	4.5	4.9	5.7	7.2	9.0	11.6
Jilin	2.4	0.4	0.4	4.0	0.0	4.1	3.9	4.2	4.6	6.2	5.6
Heilongjiang	101.8	78.6	70.1	144.0	131.7	208.2	295.7	278.4	291.8	238.1	232.7
Shanghai	21.0	35.6	47.3	46.7	46.6	58.0	62.9	52.7	62.5	45.9	39.5
Jiangsu	2412.8	2436.8	2410.7	2374.1	2344.3	2304.4	2245.8	2200.2	2145.2	2117.0	2039.3
Zhejiang	103.7	85.3	99.0	89.5	81.5	79.5	76.7	69.1	62.4	55.5	49.8
Anhui	2822.8	2887.6	2858.0	2802.5	2801.2	2733.9	2681.1	2619.2	2605.8	2484.4	2448.0
Fujian	0.2	0.2	0.3	0.4	0.5	0.7	0.9	1.5	1.9	2.8	3.6
Jiangxi	14.5	14.4	12.9	12.7	12.6	12.7	11.5	10.8	10.0	10.2	11.0
Shandong	4083.9	4068.0	4034.8	3924.8	3831.4	3759.3	3703.4	3648.7	3609.8	3567.9	3540.3
Henan	5714.6	5704.9	5623.1	5581.2	5518.0	5468.8	5430.1	5364.6	5326.4	5302.0	5234.1
Hubei	1153.2	1140.7	1122.2	1099.4	1117.1	1084.1	1028.3	1011.7	1002.0	1006.4	1099.4
Hunan	28.3	22.8	34.1	34.9	36.2	38.9	43.8	41.9	29.8	14.1	13.8
Guangdong	0.5	0.9	0.9	0.9	0.9	0.9	1.0	0.9	0.8	0.8	1.0
Guangxi	3.1	3.2	2.7	0.8	1.1	1.0	1.1	3.2	3.3	3.2	3.6
Chongqing	30.1	34.3	41.1	52.0	64.7	78.8	90.5	104.5	125.8	154.4	178.3
Sichuan	652.7	684.0	746.9	814.3	878.7	934.1	998.5	1051.2	1111.5	1172.5	1257.1
Guizhou	156.0	169.2	180.4	189.1	196.2	209.7	215.5	226.1	236.2	244.4	234.2
Yunnan	343.7	344.2	356.6	369.4	391.7	403.2	417.3	416.8	423.6	420.2	423.0
Shaanxi	963.2	980.8	1002.6	1000.6	1021.7	1078.7	1089.2	1119.7	1119.2	1117.7	1133.4
Gansu	766.5	774.7	806.4	802.8	820.9	842.0	868.6	885.3	968.5	906.4	983.7
Qinghai	82.6	84.7	82.8	80.2	84.7	86.1	90.9	96.0	95.7	98.4	99.6
Ningxia	123.1	117.3	122.5	127.5	148.8	179.0	202.1	211.4	218.5	204.3	233.7
**Province**	**2006**	**2005**	**2004**	**2003**	**2002**	**2001**	**2000**	**1999**	**1998**	**1997**	**1996**
Beijing	63.1	53.3	39.2	35.8	47.4	72.6	121.7	168.0	171.2	171.3	171.2
Tianjin	103.4	98.9	79.0	78.3	95.9	106.7	121.7	143.2	153.4	151.1	147.6
Hebei	2504.5	2377.1	2161.5	2192.9	2449.6	2579.8	2678.8	2729.9	2764.0	2720.7	2591.2
Shanxi	659.6	721.0	648.9	720.6	798.1	820.6	893.2	919.2	963.4	951.2	940.4
Liaoning	8.0	22.3	20.6	20.1	49.8	98.8	117.6	153.0	150.2	167.9	177.9
Jilin	1.1	9.5	11.4	22.1	23.0	53.8	77.3	67.5	74.5	63.5	76.6
Heilongjiang	243.5	248.5	255.0	229.6	260.8	423.3	590.2	953.4	961.4	1074.4	1231.4
Shanghai	31.4	29.9	21.9	21.7	31.4	32.0	57.1	97.2	103.9	83.3	65.3
Jiangsu	1912.7	1684.4	1601.2	1620.5	1715.9	1712.8	1954.6	2251.7	2315.0	2341.4	2216.3
Zhejiang	45.4	67.1	59.5	71.5	94.2	121.4	177.6	257.9	255.1	245.2	222.3
Anhui	2307.8	2108.3	2059.9	2012.0	2056.9	1961.2	2126.4	2057.1	2095.0	2137.6	2065.8
Fujian	4.9	5.9	6.2	8.8	23.5	30.4	38.7	50.2	55.0	60.3	64.1
Jiangxi	11.8	15.9	19.1	20.6	28.5	38.3	51.4	61.5	65.8	73.5	72.0
Shandong	3556.6	3278.7	2968.2	3105.1	3397.5	3545.8	3748.2	4006.8	3982.0	4037.6	4031.6
Henan	5208.5	4962.7	4856.0	4804.6	4855.7	4801.6	4922.3	4884.6	4964.0	4927.3	4868.2
Hubei	1016.9	716.2	602.9	603.2	700.1	735.9	845.1	1074.4	1211.2	1276.5	1230.1
Hunan	13.5	65.7	76.2	86.3	99.8	110.0	118.6	129.7	144.6	163.1	170.4
Guangdong	1.2	6.5	6.0	5.8	10.7	11.2	13.7	15.2	17.8	19.4	22.5
Guangxi	3.9	10.7	11.7	12.3	12.8	14.7	19.5	19.8	25.5	31.9	25.2
Chongqing	164.8	279.7	280.5	322.7	388.2	422.1	466.2	531.6	548.2	556.3	545.4
Sichuan	1287.2	1262.3	1255.7	1318.7	1456.9	1498.6	1605.0	1818.3	1864.6	1824.5	2364.9
Guizhou	243.9	410.6	429.2	474.3	498.4	520.5	567.4	596.3	604.5	596.6	584.2
Yunnan	437.7	532.3	543.3	567.4	604.2	640.7	645.6	724.9	706.8	697.5	664.3
Shaanxi	1159.3	1211.5	1152.7	1233.3	1356.7	1424.2	1537.2	1589.5	1610.5	1602.8	1597.8
Gansu	958.5	1000.8	933.5	961.3	1080.0	1124.0	1192.2	1222.7	1323.5	1320.1	1352.4
Qinghai	151.8	96.8	102.2	107.0	142.5	156.2	165.6	182.9	211.9	213.5	210.6
Ningxia	250.3	276.0	279.0	319.3	370.8	299.3	292.6	267.5	316.8	312.2	313.9

**Table A5:** Corn planting area in each province from 1996 to 2017 (unit: thousands of hectares).

Province	2017	2016	2015	2014	2013	2012	2011	2010	2009	2008	2007
Beijing	49.7	64.3	76.3	88.6	114.5	132.0	140.5	149.8	150.8	146.2	139.0
Tianjin	201.4	219.5	215.7	203.6	192.3	179.9	169.4	169.3	166.2	159.9	162.3
Hebei	3544.1	3696.1	3654.4	3542.1	3428.5	3323.2	3264.7	3191.0	3080.4	2885.5	2903.2
Shanxi	1806.9	1860.7	1894.5	1868.6	1836.3	1810.4	1762.2	1635.3	1511.5	1416.4	1287.8
Liaoning	2692.0	2789.8	2922.4	2758.7	2603.1	2504.6	2372.2	2277.4	2092.5	1966.2	2041.2
Jilin	4164.0	4242.0	4251.1	4062.6	3808.2	3534.2	3340.2	3215.0	3029.5	2987.6	2885.4
Heilongjiang	5862.8	6528.4	7361.2	6707.8	6571.2	6100.5	5179.7	4756.2	4361.6	3849.4	4055.4
Shanghai	3.0	4.0	4.3	4.9	4.4	4.5	4.8	4.9	4.5	3.8	4.0
Jiangsu	543.2	540.2	541.0	519.7	467.5	453.9	448.2	439.6	433.8	432.7	393.1
Zhejiang	51.9	49.9	51.6	51.1	50.3	50.8	26.2	23.9	24.5	24.3	22.9
Anhui	1160.1	1203.3	1206.3	1098.7	1045.3	975.0	952.9	864.1	803.7	731.3	733.3
Fujian	26.8	26.2	27.8	28.6	29.7	30.1	30.2	30.5	30.8	32.2	32.4
Jiangxi	35.7	35.6	31.8	28.2	26.0	25.4	24.3	24.1	21.0	16.0	14.6
Shandong	4000.1	4059.3	3943.8	3828.6	3663.1	3476.6	3370.6	3247.5	3131.1	3013.0	2855.6
Henan	3998.9	4210.5	4189.9	4009.4	3823.6	3564.7	3398.4	3233.5	3104.9	2954.4	2844.7
Hubei	794.8	797.3	813.5	745.7	653.4	663.6	603.4	572.5	536.5	488.2	444.6
Hunan	365.8	370.5	366.9	361.9	358.4	354.0	336.6	299.8	286.9	244.1	221.5
Guangdong	121.0	123.8	127.2	130.8	135.4	137.4	143.2	139.4	148.8	132.9	127.9
Guangxi	591.2	603.3	617.0	579.3	583.5	577.0	563.0	536.5	533.0	488.7	490.0
Chongqing	447.3	453.9	451.9	450.8	451.7	457.5	455.8	452.9	452.3	451.0	451.4
Sichuan	1863.9	1866.0	1816.9	1739.1	1685.8	1629.8	1574.3	1520.9	1454.8	1402.3	1369.4
Guizhou	1006.4	1041.6	1037.8	1034.8	988.5	951.4	934.5	895.5	832.6	786.6	756.6
Yunnan	1763.8	1784.8	1762.6	1745.8	1703.5	1623.1	1559.3	1527.5	1444.8	1384.7	1309.6
Shaanxi	1196.9	1341.8	1203.9	1212.8	1226.0	1241.6	1252.7	1257.5	1219.1	1193.8	1171.9
Gansu	1041.0	1056.7	1065.0	1045.4	1014.0	932.6	861.8	853.9	668.6	563.3	494.7
Qinghai	18.9	20.1	21.3	21.5	19.1	19.3	17.8	11.0	4.8	2.0	0.8
Ningxia	306.3	313.2	301.8	288.8	262.0	245.9	231.1	223.4	215.1	208.5	206.0
**Province**	**2006**	**2005**	**2004**	**2003**	**2002**	**2001**	**2000**	**1999**	**1998**	**1997**	**1996**
Beijing	135.8	119.7	93.5	75.2	87.2	100.1	135.8	198.1	207.7	206.3	207.8
Tianjin	150.9	138.8	134.8	124.9	146.5	140.9	131.2	168.6	163.0	152.2	162.9
Hebei	2799.9	2677.4	2630.6	2488.8	2577.4	2543.4	2478.6	2663.8	2581.0	2425.9	2524.9
Shanxi	1260.4	1183.7	1125.6	915.5	891.0	837.8	793.7	923.0	886.6	822.8	836.6
Liaoning	1983.1	1792.5	1598.8	1434.9	1431.6	1566.8	1422.5	1677.8	1638.0	1573.4	1576.7
Jilin	2880.7	2775.2	2901.5	2627.2	2579.5	2609.5	2197.3	2375.5	2421.3	2454.2	2481.3
Heilongjiang	3305.1	2220.2	2179.5	2053.8	2285.6	2132.7	1801.3	2651.9	2487.2	2544.8	2663.7
Shanghai	3.9	4.3	4.2	4.6	4.5	5.2	5.2	7.3	7.3	6.8	8.3
Jiangsu	378.2	370.2	389.1	451.9	436.5	429.8	423.2	454.3	473.5	439.0	467.8
Zhejiang	22.0	62.9	54.5	51.9	52.2	51.8	52.2	46.8	43.8	42.1	38.8
Anhui	623.2	670.2	662.3	627.4	651.4	589.3	485.9	588.5	570.3	512.2	614.8
Fujian	33.3	39.1	37.8	36.9	36.2	35.4	36.8	36.7	35.5	32.7	31.6
Jiangxi	14.8	16.5	14.4	17.5	16.8	19.9	25.3	27.3	32.7	31.0	40.2
Shandong	2844.4	2731.4	2455.1	2405.9	2530.1	2505.2	2413.9	2768.2	2781.9	2626.8	2826.7
Henan	2751.7	2508.3	2420.0	2386.7	2319.9	2200.0	2201.3	2193.7	2152.7	1952.4	2150.2
Hubei	431.9	389.6	357.5	341.1	390.8	400.9	424.1	460.8	440.9	399.8	405.1
Hunan	196.1	277.3	276.5	289.8	272.9	269.8	278.5	280.1	221.8	171.8	163.3
Guangdong	118.8	136.7	137.9	135.7	141.9	164.6	189.3	177.7	156.1	133.6	103.7
Guangxi	516.3	575.7	586.6	531.1	520.3	556.9	610.7	594.0	578.7	561.1	558.5
Chongqing	440.5	460.3	460.4	455.5	476.9	489.9	500.6	519.9	526.1	513.2	519.7
Sichuan	1291.7	1196.6	1172.6	1161.3	1207.9	1200.8	1235.5	1359.2	1364.8	1290.4	1762.1
Guizhou	734.9	719.5	706.5	686.3	703.8	721.8	727.3	725.6	728.8	629.5	636.0
Yunnan	1251.2	1182.6	1111.1	1066.9	1128.9	1138.1	1129.7	1159.6	1095.7	979.5	993.8
Shaanxi	1129.8	1097.1	1047.4	948.3	999.9	1005.1	1057.0	1123.4	1065.2	915.9	1087.4
Gansu	517.7	484.8	487.7	490.5	503.5	467.1	464.4	531.2	511.7	486.2	430.5
Qinghai	1.9	1.1	1.6	0	1.8	2.3	2.1	2.5	2.3	0	0
Ningxia	182.5	178.3	187.9	176.3	155.1	147.8	131.1	162.7	143.2	131.8	121.5

**Table A6:** The number and incidence of influenza in each province from 2004 to 2017 (unit: thousands of hectares).

Province	2017		2016		2015		2014		2013		2012		2011	
	N	I	N	I	N	I	N	I	N	I	N	I	N	I
Beijing	37,439	172.2997	20,279	93.4301	3439	15.9835	10,376	49.0637	2368	11.4435	1003	4.9688	391	1.9936
Tianjin	6149	39.3632	2387	15.4304	1001	6.5994	2313	15.7111	631	4.4652	1004	7.4119	415	3.2075
Hebei	39,054	52.2752	28,814	38.8072	22,537	30.5224	25,054	34.1679	21,082	28.9289	20,734	28.6361	16,423	22.8559
Shanxi	7463	20.2709	7484	20.4251	6232	17.0835	8109	22.3401	5954	16.4893	6209	17.2795	1584	4.4355
Liaoning	2031	4.6393	2026	4.623	1498	3.4115	1751	3.9886	1313	2.9916	898	2.0488	242	0.5532
Jilin	997	3.648	878	3.1889	645	2.3434	1063	3.8637	584	2.1233	886	3.2225	266	0.9686
Heilongjiang	1318	3.4693	1052	2.7597	431	1.1244	796	2.0756	154	0.4017	454	1.1841	267	0.6969
Shanghai	6215	25.685	4771	19.7535	6031	24.8631	4872	20.1727	2120	8.9060	4034	17.1845	1315	5.7126
Jiangsu	10,113	12.6435	5218	6.5419	4102	5.1532	3998	5.0356	2450	3.0934	2809	3.5562	1006	1.2789
Zhejiang	30,434	54.4437	14,394	25.9866	7970	14.4699	9700	17.6428	3302	6.0288	2903	5.3139	1995	3.6655
Anhui	19,572	31.5904	14,451	23.522	11,256	18.5043	9652	16.0072	5983	9.9917	5660	9.4839	3264	5.4857
Fujian	9625	24.8451	10,332	26.9133	8236	21.6395	8503	22.5305	4775	12.7401	4571	12.2876	2278	6.1744
Jiangxi	12,868	28.0211	9462	20.7245	8377	18.4428	8036	17.7703	5773	12.8178	5222	11.6344	3845	8.6273
Shandong	11,200	11.2601	7187	7.2986	4919	5.0248	6153	6.3215	3877	4.0032	4148	4.3041	2313	2.4146
Henan	20,418	21.4196	22,148	23.3629	17,522	18.5693	15,639	16.6136	13,505	14.3579	11,018	11.7363	6262	6.6595
Hubei	35,767	60.7766	11,610	19.8411	9201	15.8202	5065	8.7343	3469	6.0028	5474	9.5068	3113	5.4387
Hunan	27,597	40.4529	15,874	23.4289	8705	12.9207	10,617	15.8685	8136	12.2550	6593	9.9961	5256	8.0020
Guangdong	110,879	100.8084	84,209	77.6189	46,219	43.0987	50,788	47.7151	17,327	16.3555	12,947	12.3246	4599	4.4093
Guangxi	19,633	40.5818	8595	17.9212	4464	9.3900	5438	11.5236	3043	6.4994	2474	5.3262	1241	2.6963
Chongqing	5434	17.8256	3152	10.449	2357	7.8793	2084	7.0168	1966	6.6757	2560	8.7701	1152	3.9936
Sichuan	7104	8.5984	3991	4.8647	2543	3.1240	2171	2.6779	1971	2.4405	2455	3.0497	1467	1.8242
Guizhou	3946	11.0999	3419	9.6869	3329	9.4897	2274	6.493	2781	7.9822	1834	5.2868	878	2.5269
Yunnan	3496	7.3276	2656	5.6012	1958	4.1537	1736	3.7042	2839	6.0936	2071	4.4720	971	2.1124
Shaanxi	12,076	31.6695	5975	15.7532	3592	9.5149	5344	14.1977	4936	13.1518	4218	11.2702	1348	3.6113
Gansu	7296	27.954	8479	32.6172	5056	19.5154	6344	24.5684	4916	19.0724	5194	20.2559	2724	10.6509
Qinghai	1226	20.6746	767	13.0347	276	4.7307	707	12.2363	487	8.4966	254	4.4705	148	2.6303
Ningxia	1475	21.8551	1434	21.4709	925	13.9825	1469	22.4553	1030	15.9150	1416	22.1440	444	7.0461
**Province**	**2010**		**2009**		**2008**		**2007**		**2006**		**2005**		**2004**	
	**N**	**I**	**N**	**I**	**N**	**I**	**N**	**I**	**N**	**I**	**N**	**I**	**N**	**I**
Beijing	830	4.7293	5147	30.3658	335	2.0514	221	1.3978	290	1.8855	120	0.7811	8	0.0540
Tianjin	523	4.2584	707	6.0119	597	5.3542	831	7.7302	1676	16.0690	377	3.6197	13	0.1399
Hebei	13,679	19.4459	15,081	21.5786	11,615	16.7291	8679	12.5819	7117	10.3883	3916	5.7508	1923	2.8283
Shanxi	787	2.2962	2456	7.2011	172	0.5069	265	0.7852	721	2.1490	79	0.2369	57	0.1720
Liaoning	1240	2.8710	775	1.7962	62	0.1443	129	0.3020	89	0.2109	66	0.1565	4	0.0096
Jilin	717	2.6172	343	1.2546	149	0.5458	167	0.6133	50	0.1841	30	0.1107	4	0.0150
Heilongjiang	537	1.4036	1407	3.6785	84	0.2197	52	0.1360	24	0.0628	65	0.1706	17	0.0453
Shanghai	2429	12.6444	1391	7.3658	269	1.4478	404	2.2259	77	0.4331	8	0.0450	15	0.1121
Jiangsu	2267	2.9346	5255	6.8449	575	0.7541	1969	2.6079	4658	6.2314	1028	1.3783	370	0.4889
Zhejiang	3257	6.2876	7288	14.2344	894	1.7668	936	1.8795	4298	8.7750	1998	4.0997	3780	8.0430
Anhui	2664	4.3451	3490	5.6887	675	1.1033	925	1.5139	1431	2.3382	1097	1.8036	744	1.1547
Fujian	2101	5.7927	6041	16.6301	884	2.4686	361	1.0146	614	1.7369	477	1.3574	685	1.9051
Jiangxi	2550	5.7534	6257	14.2205	1680	3.8461	1093	2.5189	2039	4.7298	1266	2.9597	1497	3.4948
Shandong	3075	3.2470	4559	4.8411	429	0.4580	292	0.3137	163	0.1763	96	0.1044	157	0.1712
Henan	3936	4.1488	8849	9.3849	3594	3.8397	2710	2.8854	2991	3.1887	1885	2.0201	772	0.7965
Hubei	1994	3.4860	15,444	27.0425	1834	3.2181	1802	3.1653	1565	2.7408	1205	2.1168	159	0.2646
Hunan	4390	6.8529	19,514	30.5633	3621	5.6979	1918	3.0243	894	1.4132	664	1.0550	1010	1.5121
Guangdong	5957	6.1807	20,155	21.1180	3334	3.5284	2800	3.0095	8070	8.7775	5913	6.4765	4973	6.2520
Guangxi	1233	2.5391	11,969	24.8526	955	2.0029	931	1.9729	1999	4.2897	3234	6.9965	8605	17.7209
Chongqing	960	3.3578	11,640	41.0004	1787	6.3459	669	2.3825	1161	4.1494	2343	8.3989	3351	10.7057
Sichuan	1530	1.8693	9517	11.6308	1707	2.1004	1732	2.1202	2054	2.5012	5157	6.2981	12,560	14.4138
Guizhou	1256	3.3070	10,472	27.6398	1612	4.2850	1063	2.8293	1764	4.7292	6838	18.4677	5583	14.6039
Yunnan	723	1.5817	7664	16.8699	395	0.8751	435	0.9703	4487	10.0833	3753	8.5011	3	0.0069
Shaanxi	640	1.6967	6287	16.7119	346	0.9232	348	0.9317	354	0.9516	342	0.9230	1050	2.8703
Gansu	2276	8.6361	7966	30.3106	2522	9.6369	3689	14.1557	4212	16.2374	1110	4.3049	978	3.7416
Qinghai	97	1.7405	211	3.8066	47	0.8515	103	1.8796	177	3.2597	190	3.5323	230	4.2627
Ningxia	1348	21.5610	2870	47.3922	684	11.2132	547	9.0563	315	5.2852	265	4.4951	193	3.3051

Note: N = Number; I = Incidence.

**Table A7:** Per capita GDP of each province from 1996 to 2017 (unit: yuan).

Province	2017	2016	2015	2014	2013	2012	2011	2010	2009	2008	2007
Beijing	137,596	124,516	114,662	107,472	101,023	93,078	86,365	78,307	71,059	68,541	63,629
Tianjin	79,837	73,830	71,021	71,198	68,937	65,346	61,137	54,053	47,497	45,242	37,976
Hebei	40,883	38,233	35,653	34,260	33,187	31,770	29,631	25,308	21,831	20,385	17,561
Shanxi	39,232	32,526	32,375	33,237	33,111	32,435	30,400	25,434	20,906	21,234	17,542
Liaoning	49,603	46,557	46,069	45,608	43,758	40,694	37,350	31,888	29,611	28,185	24,022
Jilin	40,077	38,011	36,391	36,218	34,273	31,558	28,146	23,370	19,858	17,696	14,966
Heilongjiang	32,454	31,258	30,583	31,744	30,901	28,732	25,915	21,694	18,871	18,654	16,023
Shanghai	13,6109	12,3628	11,1081	10,4402	96,773	90,127	86,061	79,396	72,363	69,154	63,951
Jiangsu	10,7150	96,840	89,426	81,550	74,844	67,896	61,947	52,787	44,272	39,967	33,798
Zhejiang	93,186	84,921	78,768	72,730	68,036	62,856	58,398	51,110	43,543	41,061	36,454
Anhui	47,671	42,641	38,983	37,184	34,256	30,683	27,314	21,923	17,715	15,535	12,989
Fujian	86,943	76,778	70,162	65,810	59,835	54,073	48,341	40,773	33,999	30,153	25,915
Jiangxi	43,868	40,159	36,850	34,571	31,686	28,486	25,885	21,099	17,277	15,816	13,270
Shandong	63,162	59,375	56,312	52,016	48,763	44,464	40,639	35,599	31,282	28,861	24,329
Henan	46,959	42,341	39,209	36,686	33,618	30,820	28,009	23,984	20,280	18,879	15,811
Hubei	63,180	56,836	52,015	48,630	43,838	39,163	34,738	28,359	23,081	20,153	16,593
Hunan	49,448	45,356	42,216	38,549	35,328	32,048	28,734	24,005	19,979	17,758	14,626
Guangdong	82,686	75,213	69,283	63,809	58,860	54,038	50,676	44,669	39,418	37,543	33,236
Guangxi	36,595	33,458	30,990	28,687	26,483	24,238	22,258	18,070	14,708	13,471	11,542
Chongqing	65,538	59,433	53,398	49,062	44,049	39,548	35,017	28,084	23,346	20,865	16,966
Sichuan	45,768	40,251	37,129	35,565	32,772	29,669	26,163	21,230	17,387	15,685	12,963
Guizhou	38,137	33,291	29,956	26,171	22,825	19,394	16,165	12,882	10,814	9697	7778
Yunnan	38,629	34,416	31,642	29,874	27,447	23,891	20,629	16,866	14,427	13,286	11,287
Shaanxi	56,154	50,081	47,301	46,167	42,318	37,733	32,562	26,388	21,485	19,331	15,342
Gansu	28,026	26,520	25,264	25,202	23,313	20,978	18,801	15,421	12,802	12,048	10,501
Qinghai	41,366	38,213	34,322	31,824	29,772	26,784	24,220	20,418	16,907	16,220	13,100
Ningxia	47,177	41,427	38,805	37,605	35,772	33,125	30,365	24,984	20,382	18,554	14,458
**Province**	**2006**	**2005**	**2004**	**2003**	**2002**	**2001**	**2000**	**1999**	**1998**	**1997**	**1996**
Beijing	53,438	47,182	42,402	36,583	32,231	28,097	25,014	22,054	19,625	16,949	14,495
Tianjin	33,411	30,567	25,761	22,371	19,161	17,523	16,236	14,985	14,086	13,142	11,734
Hebei	14,609	12,845	11,178	9380	8216	7572	6966	6310	5994	5615	4950
Shanxi	14,008	12,195	10,515	8639	7082	6226	5722	5230	5104	4724	4178
Liaoning	19,760	17,210	15,355	14,041	13,000	12,015	11,177	10,086	9415	8725	7730
Jilin	11,864	10,237	9073	7925	7581	7076	6646	6311	5983	5591	5178
Heilongjiang	13,947	12,456	10,836	9464	8507	7990	7515	6707	6566	6412	5755
Shanghai	54,996	49,377	44,998	39,117	34,277	32,089	30,307	27,293	25,405	23,573	20,808
Jiangsu	27,868	23,984	19,790	16,743	14,369	12,879	11,765	10,695	10,049	9371	8471
Zhejiang	30,415	26,277	23,476	20,249	16,918	14,726	13,467	12,229	11,395	10,615	9534
Anhui	10,630	9193	8279	7001	6238	5732	5147	4819	4516	4160	3703
Fujian	20,915	18,107	16,248	14,330	12,910	11,883	11,194	10,323	9603	8775	7658
Jiangxi	10,859	9172	7960	6636	5829	5221	4851	4402	4124	3890	3452
Shandong	20,443	17,308	14,540	11,977	11,120	10,063	9260	8483	7968	7461	6746
Henan	12,761	10,978	9047	7376	6487	5959	5450	4832	4643	4389	3978
Hubei	13,210	11,342	9746	8378	7437	6866	6121	5452	5287	4884	4311
Hunan	11,733	10,200	9004	7589	6734	6120	5590	4933	4667	4420	3963
Guangdong	27,861	23,997	20,647	17,950	15,478	13,952	12,817	11,463	10,850	10,154	9157
Guangxi	9421	8069	7182	6120	5559	5058	4652	4444	4346	3928	3706
Chongqing	13,915	12,335	10,934	9311	8079	7096	6383	5890	5649	5306	4613
Sichuan	10,371	8828	7751	6565	5890	5376	4956	4540	4294	4032	3550
Guizhou	6103	5218	4244	3708	3257	3000	2759	2545	2364	2250	2048
Yunnan	9158	7890	7136	6048	5472	5063	4814	4558	4446	4121	3779
Shaanxi	12,439	10,357	8545	7057	6161	5511	4968	4415	4070	3834	3446
Gansu	8653	7332	6512	5525	4875	4467	4163	3778	3541	3199	2946
Qinghai	10,728	9233	8275	7248	6478	5774	5138	4728	4425	4122	3799
Ningxia	11,389	9796	8904	7686	6647	6039	5376	4900	4607	4277	3926

**Table A8:** The number of domestic tourists received by each province from 2013 to 2017 (unit: 100 million person-times).

Province	2017	2016	2015	2014	2013
Beijing	2.9	2.8	2.6	2.6	2.5
Shanghai	1.55	1.47	1.39	1.3	1.13
Guangdong	4.07	3.62	3.28	2.93	2.67
Tianjin	2	1.8	1.7	1.5	1.36
Jiangsu	7.43	6.78	6.19	5.7	5.2
Zhejiang	6.4	5.73	5.25	4.79	4.34
Liaoning	5.03	4.49	3.97	4.59	4.04
Shandong	7.7	7	6.5	5.9	5.4
Fujian	3.75	3.09	2.61	2.29	1.95
Sichuan	6.7	6.3	5.9	5.4	4.9
Hebei	5.7	4.7	3.7	3.1	2.7
Hubei	6.39	5.73	5.07	4.69	4.06
Henan	6.6	5.8	5.1	4.5	4
Hunan	6.7	5.6	4.7	4.1	3.6
Heilongjiang	1.63	1.44	1.3	1.05	2.9
Chongqing	5.2	4.5	3.9	3.4	2.9
Jilin	0.5	0.43	0.38	0.32	0.27
Jiangxi	5.7	4.6	3.8	3.1	2.4
Shanxi	5.6	4.4	3.6	3	2.5
Shaanxi	5.19	4.46	3.83	3.29	2.82
Anhui	6.26	5.22	4.44	3.8	3.36
Yunnan	5.67	4.25	3.23	2.81	2.4
Guangxi	5.18	4	3.3	2.8	2.4
Gansu	2.38	1.9	1.56	1.26	1
Guizhou	6.7	5.3	3.75	3.2	2.6
Ningxia	0.3	0.2	0.18	0.16	0.18
Qinghai	0.34	0.28	0.23	0.2	0.18

**Table A9:** Travel index of each province in 2013–2017.

Province	2017	2016	2015	2014	2013
Jiangsu	3	4	4	4	3
Guangdong	4	3	3	2	4
Zhejiang	5	5	5	5	5
Shanghai	1	1	2	3	2
Shandong	6	6	7	6	6
Beijing	2	2	1	1	1
Henan	10	13	12	12	11
Sichuan	14	15	14	15	12
Fujian	7	8	9	9	9
Anhui	16	16	17	13	16
Hunan	9	10	13	14	14
Hebei	12	9	11	11	13
Shaanxi	17	17	15	16	15
Hubei	11	12	10	10	10
Liaoning	13	11	8	8	8
Chongqing	15	14	16	19	17
Tianjin	8	7	6	7	7
Heilongjiang	21	18	18	18	18
Shanxi	18	19	19	23	19
Yunnan	22	24	26	26	25
Jiangxi	20	22	21	20	21
Guangxi	25	25	24	24	24
Jilin	23	20	20	22	20
Guizhou	26	26	29	29	30
Gansu	28	28	28	28	28
Ningxia	29	29	27	27	27
Qinghai	30	30	30	30	29
